# ‘Empowerment’ as a proximal implementation outcome for task shifting with informal cadres: findings from a qualitative study with traditional healers in rural Uganda

**DOI:** 10.1186/s43058-025-00823-9

**Published:** 2025-11-29

**Authors:** Misha Hooda, Madison Stead, Gabriel Nuwagaba, Sylvia Natukunda, Constance Birungi, William Bugeza, Maureen Tushabe, Srija Gogineni, Denis Nansera, Winnie Muyindike, Juliet Mwanga-Amumpaire, Radhika Sundararajan

**Affiliations:** 1 Weill Cornell Medicine (WCM), 1300 York Avenue, New York, NY 10021 USA; 2 Cornell University, 410 Thurston Avenue, Ithaca, NY 14850 USA; 3 Mbarara University of Science and Technology (MUST), P.O BOX 1410, Mbarara, Uganda; 4 Weill Cornell Medicine - Center for Global Health, 402 E. 67th Street, New York, NY 10065 USA

**Keywords:** Medical pluralism, Task-sharing, Lay cadre, Africa, Implementation outcomes, Health systems research

## Abstract

**Background:**

Task shifting and task sharing (TSS) are widely used implementation strategies to expand HIV service delivery in low-resource settings. Informal lay health workers, such as traditional healers (THs), have been proposed as critical partners in bridging service delivery gaps. However, the mechanisms that support their successful integration into formal health systems remain underexplored. This qualitative sub-study aimed characterizes a novel proximal implementation outcome – empowerment – based on lived experiences of THs participating in a TSS intervention in rural Uganda.

**Methods:**

Between July and August 2023, we conducted 22 in-depth interviews with THs in rural Uganda who completed a three day training to become lay HIV supporters. The curriculum included HIV transmission, ART adherence, stigma reduction, and HIV self-testing. Interviews were conducted in the local language, transcribed, translated into English, and analyzed using a thematic approach. Our analysis was guided by Lee and Koh’s empowerment framework, which links role transformation to domains of empowerment.

**Results:**

THs reported experiencing empowerment across four domains: meaningfulness, competence, self-determination, and impact. Participants described strong alignment between their traditional caregiving roles and new responsibilities in HIV support. They reported increased HIV-related knowledge, confidence in client care, autonomy in decision-making, and a sense of contributing meaningfully to improved health outcomes. Notably, we identified a fifth domain – external validation – defined as recognition and legitimacy conferred by representatives of the biomedical health system. This domain was central to participants’ perceived integration, motivation, and potential sustainability of their involvement in these types of programs.

**Conclusions:**

We propose empowerment as a novel proximal implementation outcome that reflects the internal and external transformations necessary for successful implementation with informal providers. Our findings support expanding Lee and Koh’s empowerment framework to include external validation, particularly for cadres operating outside the formal system. Positioning empowerment as a proximal outcome offers a valuable lens for evaluating early success of broad implementation strategies that involve role transformation, such as training trainers, or engaging community champions.

**Trial registration:**

ClinicalTrials.gov, NCT05943548. Registered 2023–07-13, https://clinicaltrials.gov/study/NCT05943548.

Contributions to the Literature

- Task-shifting to community health workers has been widely implemented to address workforce shortages, but implementation with lay cadres remain understudied. Therefore, little is known about potentiating and evaluating successful engagement with these important cadres.
- This study extends Lee and Koh’s empowerment framework by identifying *external validation*—recognition from the formal healthcare system—as a crucial factor in sustaining lay worker engagement in task-shifting programs.
- This study responds to the call by Proctor et al. (2011, 2023) advance the identification of new implementation outcomes. In the 2023 agenda, Proctor et al. noted the specific need to identify measurable proximal outcomes to help understand how a strategy works to achieve what it is targeted to change.

## Introduction

As Uganda inches closer to the 95–95-95 targets established by UNAIDS, persistent gaps in HIV care continuity—particularly in rural areas—highlight the need for implementation strategies that reach underserved population [1]. Barriers such as geographic remoteness, health worker shortages, and low perceived need for biomedical care undermine progress across the HIV care cascade in rural settings [2, 3]. To address these challenges, task shifting and task sharing (TSS) have emerged as core implementation strategies designed to expand service delivery capacity without overburdening formal health systems [4–11]. TSS encompasses two complementary approaches: task shifting, which redistributes care responsibilities to less specialized providers, and task sharing, which builds capacity among additional cadres to take on identified tasks without removing them from existing roles [6, 12–14]. This implementation strategy correspond to the Expert Recommendations for Implementing Changes (ERIC) taxonomy’s implementation strategy of “revising professional roles” [15], and have become foundational to HIV care delivery in sub-Saharan Africa, where they have improved ART adherence, clinical outcomes, and service reach [8, 9, 16–22].

Despite successful implementation of community health worker (CHW) task-shifting initiatives through village health teams, significant implementation gaps persist. While these programs have positively impacted antiretroviral therapy (ART) adherence and clinic retention rates [23, 24], rural populations remain significantly under-engaged throughout the HIV care continuum when compared to their urban counterparts. Current viral suppression rates of 69.0% in rural areas versus 75.5% in urban areas indicate suboptimal implementation reach and effectiveness in rural settings [1]. Overburdened rural health systems and existing community outreach programs are unable to mitigate longstanding barriers to HIV care that plague rural populations, including distance to clinics, wait times, and HIV-related stigma [25–30]. This implementation gap has prompted policymakers to seek novel strategies for reaching rural people living with HIV who remain disconnected from formal healthcare systems.

Recent TSS initiatives have increasingly incorporated community-based lay providers to bridge this gap [31, 32]. Yet, the majority of large-scale TSS programs focus on formalized cadres —such as community health workers, peer supporters, and village health teams —who are integrated into the formal health system and benefit from established training and supervision structures. In contrast, *informal* providers, particularly traditional healers (THs), remain largely underutilized in the design and delivery of TSS interventions despite their central role in community health and their frequent contact with hard-to-reach populations [33]. In Uganda, 80% of rural populations use traditional healers, who often serve as first points of contact for healthcare needs given high accessibility due to their community presence, and whose services are used instead of or in adjunct to biomedical care services [34–36]. As such, leveraging traditional healer support to improve testing, linkage to care, and adherence to ART in rural areas has been proposed as a way to engage people living with HIV (PLWH), who may have splintered relationships with existing biomedical care systems [34, 37–40].

Our ongoing cluster randomized controlled trial (NCT05943548) evaluates the implementation of a TSS approach where THs provide psychosocial support to PLWH to improve HIV testing and retention in care in rural Uganda [41]. In these communities, THs a provide various treatments for illness, offering herbal remedies, spiritual consultations, and counseling that address both physical and social dimensions of health. Their longstanding trust within communities and holistic orientations towards health and wellness make them influential actors in shaping care-seeking behaviors [42–45]. Though TSS programming has been extensively implemented across low- and middle-income countries, research that explores how informal providers engage with TSS roles, or what factors influence their sustained participation and delivery fidelity remains poorly understood. In particular, the implementation outcomes that mediate the success of TSS among informal cadres remains limited [8, 46].

Implementation outcomes—defined as “the effects of deliberate and purposive actions to implement new treatments, practices, and services” [47] — are central to understanding how and why interventions succeed. As outlined by Proctor et al., these outcomes serve as early markers of implementation progress, indicators of success, and mediators linking strategies to clinical effects. Yet most implementation research in TSS settings has focused on outcomes such as feasibility, acceptability, or fidelity—without examining the transformative effects of TSS that may drive success or failure of these programs, particularly with lay cadres who are not already integrated into health programming [47].

While effective knowledge transfer and worker engagement strategies are recognized as crucial components of TSS programming [6, 10, 19, 48–51], very little is known about the experiences of informal providers who operate outside the scope of biomedical health systems. While THs have been the focus of prior task shifting programs, attention has not been paid to their experiences with the task shifting work. For example, how might they integrate new responsibilities with their traditional roles, and what facilitates their sustained involvement in health programs? THs’ marginal position in the health system, distinct identities, and community roles may shape how they perceive and adapt to new expectations in ways that differ from formal health workers such as community health workers [10, 52].

We conducted a qualitative sub-study nested within our ongoing TSS programming trial (NCT05943548) to examine traditional healers' experiences with task shifting training and role transformation. The primary objective was to characterize implementation-relevant experiences and identify factors that facilitate or impede successful TSS programming implementation among this informal lay cadre. This study contributes to the growing body of literature on task-shifting and task-sharing by providing empirical insights on TSS programming with lay health workers. We use a theory-driven approach to decompose the effects of TSS and characterize a novel proximal implementation outcome, supported by end-user perspectives.

## Methods

### Study design

This qualitative sub-study was embedded within a cluster randomized hybrid type I effectiveness-implementation trial. The overall goal of the trial is to evaluate if healers can improve HIV viral suppression through delivery of adherence and psychosocial support to PLWH in communities in the Mbarara and Rwampara Districts of southwestern Uganda [41].

To ensure that intervention arm healers were adequately trained to complete the tasks of HIV testing, pre- and post-test counselling, facilitating linkage to HIV care, and providing psychosocial support to PLWH, THs completed a three day training course in July 2023, prior to trial initiation. The TSS curriculum for Ugandan healers was developed and pilot-tested in 2022 [43] and was adapted to meet the knowledge requirements of the parent trial. Curriculum included education on HIV transmission and prevention, training on ART adherence support, facilitating serostatus disclosure, and information on clinical HIV services.

This study was conducted following ethical standards and was approved by the Mbarara University of Science and Technology Research Ethics Committee (MUST-2022–646), the Weill Cornell Medicine Research Ethics Committee (22–09025268), and the Uganda National Council for Science and Technology (HS2780ES). All participants provided written informed consent prior to participation in both the parent trial as well as this qualitative sub-study.

### Study population

All twenty-two traditional healers enrolled in the parent study intervention arm at the time of the TSS training were invited to participate in a single one-on-one, semi-structured, in-depth qualitative interview to explore their views of the study and their lived experiences following training. This census-based sampling ensured full representation of the intervention cadre and supported thematic saturation.

### Data collection

Qualitative interviews lasted approximately 60 min and explored various constructs guided by the Consolidated Framework for Implementation Research (CFIR) framework (intervention characteristics, outer setting, inner setting, individual characteristics, and implementation process) [53]. The CFIR framework was used to guide development of the interview guide to ensure that potential implementation determinants were evaluated in a rigorous and comprehensive manner. The interviews were conducted in private locations in Runyankole (the local language) by trained members of the Ugandan qualitative research team (GN, MT, CB, EA) who had no prior relationship with the participating TH prior to study commencement. Training sessions on qualitative research methods and implementation science were conducted to enhance qualitative interviewing skills among the research team. All members of the study team have considerable research experience conducting community-based healthcare and HIV service delivery.

All interviews followed interview guides, which were initially developed in English, translated into Runyankole, and then translated back to English to maintain fidelity of meaning. Interview guides were used to ensure consistency of topics covered in each interview while allowing the interviewer to explore novel concepts in real time. All interviews were audio-recorded and concurrently translated and transcribed into English by the interviewer. The study Qualitative Data Coordinator (author GN), fluent in Runyankole and English, also reviewed the English transcripts against the audio recordings to verify translation accuracy and integrity of meaning. To ensure data security, all audio files and transcripts were stored on encrypted, password-protected institutional servers accessible only to authorized study personnel. Transcripts were de-identified and pseudonyms used in all analyses and reports to maintain participant confidentiality.

### Data analysis

Employing an inductive, thematic-analysis approach, all transcripts were reviewed to develop a coding scheme to capture trial participants' experiences, organized initially within CFIR domains. In vivo codes were then independently developed by three authors (MH, MS, RS) through repeated engagement with the data set. Each author coded a subset of three transcripts individually. Coding schemas were then compared, with discrepancies in codes resolved through discussion until a consensus was reached. Using a deductive framework approach, coded data was organized by topic and entered into an analytical matrix in Microsoft Excel. Authors MH and MS reviewed the matrix to identify concepts which reflected lived experiences and perceptions of TH regarding their training and new role in HIV care programs. Our author team consisted of three male authors (GN, WB, and DN) and nine female authors (MH, MS, SN, CB, MT, SG, WM, JMA, RS), with the first author and senior author being women.

We noted congruence between our thematic analysis and Lee & Koh's empowerment framework [54] (described below), which guided subsequent deductive mapping. While Lee & Koh’s framework was originally derived from research in formal organizational settings, it has more recently been applied to evaluating CHW engagement with health programming efforts [55].

### Theoretical model

Building on Proctor et al.’s [47, 56] conceptualization, we conceive of empowerment within Lee and Koh’s framework as a *proximal implementation outcome*, or an intermediary indicator of success of an strategy along the path of implementation. Proximal outcomes are direct and measurable products of the implementation strategy and can serve as benchmarks towards achieving long term (distal) outcomes, such as adoption or sustainment [57]. Here, we describe how empowerment could be conceived as a necessary – but not sufficient – proximal outcome for successful task shifting implementation among lay workers.

Originally developed in organizational psychology, Lee & Koh's framework for worker empowerment [54] describes the four dimensions of employee empowerment as a “subordinate perceiving… meaningfulness, competence, self-determination, and impact, which is affected by empowering behaviors of a supervisor” [54].

In their model, *meaningfulness* is defined as an individual perceiving value in their task, goal, or purpose that is also tied to congruence between their beliefs and values, and the requirements of their role. *Competence* lies in an individual's belief in their ability to execute tasks effectively in their employment context. *Self-determination* refers to an individual's perceived autonomy involving decisions within their work roles. Finally, *impact* relates to an individual's perception of their ability to influence outcomes in their working environment.

Though our findings were mostly congruent with Lee and Koh’s framework, we also identified a fifth element pertaining to TH engagement, which we defined as *external validation*. This refers to an individual's perceived sense of legitimacy and acceptance within their work environment, reinforced through recognition and support from formal external structures or authorities. Unlike the other four domains, which focus on internal self-perception, external validation captures social affirmation by formal actors—especially salient for THs whose roles have historically been stigmatized or excluded from formal health services programming. This validation enhances the individual's motivation by affirming their role and contributions as integral to broader organizational or community goals. We propose this as a novel, fifth dimension of empowerment with particular relevance for implementation research involving informal providers or those from minoritzed cadres.

## Results

Twenty-two individual interviews were conducted with TH assigned to the study intervention arm. Among the 22 TH enrolled, most were female (64%) and literate or semi-literate (96%), with spiritualists being the most common specialty (45%) and the majority (68%) residing more than 5 km from the nearest HIV clinic. A more detailed summary of participant demographics is shown in Table 1.

**Table 1:** Traditional Healer Demographics (*n* = 22)

Gender	
Female	14 (64)
Literacy Level (n,%)	
Literate	12 (55)
Semi-Literate	9 (41)
Illiterate	1 (4)
Speciality (n,%)	
Herbalist	5 (23)
Traditional Birth Attendant	5 (23)
Spiritualist	10 (45)
Bonesetter	2 (9)
Age (median, IQR)	
Median Age	45 (37 - 57)
Distance from nearest HIV clinic (n,%)	
Less than 5 km	7 (32)
More than 5 km	15 (68)

Our findings indicate that following the task-shifting training, THs experienced a multidimensional sense of empowerment that enabled a significant transformation in how they understood and planned to enact their roles. Rather than simply adding new skills, the training facilitated a deeper shift—supporting THs to integrate biomedical responsibilities into their traditional caregiving identities. Participant perceptions and reactions to the training aligned with the four dimensions in Lee & Koh's employment empowerment framework (meaningfulness, competence, choice, impact). Additionally, our analysis identified a novel fifth domain, external validation, reflecting the significance of formal recognition from formal institutions in reinforcing healer legitimacy. Definitions of these five domains in the context of our study are summarized in Table 2.

**Table 2:** Key Themes

Key Theme	Description
*Meaningfulness*	TH found their roles personally significant, aligning with their cultural responsibilities and strong community ties
*Competence*	TH felt prepared to effectively and appropriately support their clients with HIV
*Self-Determination*	TH felt more autonomous, with increased capacity to make informed decisions in HIV care
*Impact*	TH recognized their role in reducing barriers to HIV care, such as stigma and inaccessibility
*External Validation*	Recognition from the formal health system reinforced TH legitimacy and motivation

### Meaningfulness

*Meaningfulness* is characterized by the personal significance TH attribute to their work and how their new TSS roles align with their intrinsic values and motivations. Healers were particularly motivated by long-standing relationships with clients and their desire to provide care where it is most needed. They expressed that the training was not only about gaining new skills but also about reinforcing their inherent role as trusted caregivers in their communities.

*They [PLWH] also believe and trust in me because of the way we have been working together as traditional healers; they know we are very good at keeping secrets. Most of these clients are the people we know; we love and care of them so much. In fact, the care that we [healers] give them is much more than that of their own parents, family, and friends. Because of that, they [PLWH] will not hesitate when it comes accepting our support for HIV care. – Female, 45 years old.*

For several participants, training reinforced their community role and introduced a new sense of professional identity that blended traditional and biomedical caregiving.

*At the moment, I look at myself more than the healer. I am like a health worker. I am so attached to my clients living with HIV because they are vulnerable and need special attention. – Female, 61 years old.*

### Competence

*Competence* in this context refers to the traditional healers’ enhanced belief in their ability to support PLWH effectively. Before the training, many healers felt constrained in their ability to provide comprehensive care due to a lack of formal HIV-related knowledge.

The training was described as helping healers overcome their initial fears in supporting PLWH due to their limited knowledge on the subject. By addressing this knowledge gap, the training fostered a new sense of professional identity by expanding their capacity to offer more comprehensive support to clients with HIV, bridging traditional and biomedical paradigms.

*I am sure that now if I got any of my clients living with HIV at my place, I feel I am the right person to help and support him or her to live a healthy life. I am not afraid anymore. I am skilled, an expert, and a professional in supporting PLWH. – Male, 38 years old.*

*After the study training, I went back home when I was fully loaded with enough information and knowledge about HIV, ready to start supporting people. As I speak, I feel like I am also a health worker because I trained. I am so blessed because I am skilled in providing services to my clients in two ways, both a traditional healer and a person providing services to PLWH. –Female, 45 years old.*

Healers also acknowledged that even though they had previously referred their clients living with HIV to clinics before receiving the training, they still gained a sense of confidence that allowed them to provide auxiliary support to biomedical care.

*The moment I discover that a client is HIV positive, I will try linking them to the hospital for ART initiation and HIV management. I also used to give the PLWH counselling, only that I had not been competent to counsel HIV clients professionally. But now, after the study training, I will be counselling my clients correctly. – Male, 32 years old.*

### Self-Determination

*Self-determination* reflects traditional healers’ increased autonomy in making decisions related to HIV care. Though healers practice outside the realm of the formal biomedical care system and have independence in individual practices, healers described limited autonomy in dealing with clients living with HIV due to lack of knowledge about the disease. Following the training, healers noted a different feeling, where they could now autonomously support their clients throughout their HIV care experience, from diagnosis through ART adherence counselling.

*Before training, I would reach a time and be handcuffed whenever I was faced with a challenging situation of an HIV-positive person. But now I am happy that I will be able to help people from the villages who would usually be left unattended and more affected by HIV and its related infections. – Male, 43 years old.*

*We used to (offer services) our best but all in vain. You would not see any improvement. You pray, but no change. Sometimes, we knew the HIV status of those clients but not on treatment, and we did not know how to support them to get back to care… This training has really changed me and opened my eyes in things related to HIV… after the training, I learnt a lot, and I am ready and prepared to do the work. – Female, 37 years old.*

Furthermore, traditional healer participants noted their appreciation for maintaining their own care practices while collaborating with the biomedical healthcare system to address HIV.

*If this project is sustained, we shall be able to rout HIV in our communities. The project should stay and stay forever because we believe that it does not negatively affect our activities as traditional healers, and yet it helps our clients living with HIV to get better. – Female, 45 years old.*

### Impact

*Impact* involves participants’ perceived ability to improve their clients' health outcomes and have a positive influence on the broader community by improving access to HIV care. Healers recognized their ability to provide crucial socio-emotional support for PLWH that formal healthcare systems often struggle to deliver, especially in rural areas. They felt that their involvement would make a significant difference by facilitating self-efficacy and encouraging PLWH to remain in formal HIV care. This further reinforced their self-concept as legitimate, capable health actors—a hallmark of role transformation.

*I have stayed with these people for a very long time. When a person comes to me, I usually know what they have been going through … People who are nearer to me, we meet at village functions and do farm work together, which makes them not fear me…The same way you told me that this is a conversation, my counselling sessions with my patients are conversations too. I ask the patients how they are doing, and when they tell me about challenges with their HIV medicines, I will advise them according to how we were trained. – Female, 57 years.*

Furthermore, healers elaborated on their ability to provide a stigma-free environment for HIV testing, counseling, and serostatus disclosure. Pre-existing, close relationships between healers and their clients was described as the foundation of this trusting relationship – a significant difference from the HIV clinical environment.

*People in villages do not usually disclose their secrets to formal health workers in health facilities, they are feared, not friendly, and very busy. They [PLWH] would rather disclose to me than health care workers.… It becomes easy to disclose to me as we talk about our usual things. They [PLWH] always see me as an adviser, since I am old and knowledgeable in my specialty. They trust me with their secrets. – Female, 70 years old.*

*It will be easier for my clients to go and test themselves from our location than going to the clinics where their HIV status will be exposed to the public. HIV testing at the healer location is good and confidential. It is private, no issues of HIV stigma and worries are not there. –Female, 37 years old.*

### External Validation

*External validation* emerged as a distinct and critical dimension of empowerment for task shifting among our participants. This theme, rooted in formal acknowledgment from members of institutional and biomedical healthcare systems, was instrumental in fostering a stronger sense of belonging, legitimacy, and motivation among our traditional healer participants. Recognition came in various forms, including physical tokens, symbolic respect, and social inclusion, each contributing significantly to healers’ enthusiasm for participating in the training and their subsequent roles in HIV care. Participants emphasized the value of receiving a tangible symbol of their new role which solidified THs’ evolving professional identities through formal recognition as valued contributors to public health efforts.

*I was also given an HIV training certificate. I am so proud of it, and when I came from the training, I showed it to my village Local Chairperson. I will be able to support my clients living with HIV, and I will not hesitate at any one time to test, counsel and link them to HIV care and treatment at the health facility. – Female, 49 years old.*

They also highlighted how inclusion in an HIV study run by local biomedical professionals bridged social gaps between traditional and biomedical practitioners, and represented a long-overdue acknowledgment of their contributions to health in their communities, addressing feelings of historical neglect:

*I appreciate the study so much for recognizing us, the traditional healers, as important people, mixing with us, equipping us with knowledge. We used fear to come near doctors, but during training, we sat with them. They come in our homes and sit with us; this showed me the love and respect you have for us [traditional healer] without discriminating against us. – Female, 55 years old.*

*For us healers, we have always been ignored and forgotten as far as health that is not concerned with spirituality is concerned…we were always ignored and left behind. I was very happy to be trained, get good connections with health workers to improve the wellbeing of our rural people. Thank you so much for thinking and remembering us. I hope it was God's plans. – Male, 57 years old.*

Each domain of empowerment reflects a distinct yet interrelated pathway through which the strategy of TSS produces engagement:

- Meaningfulness reflects perceived alignment between their identity and their newly assigned roles. Alignment is critical for role legitimacy and internal motivation, and may predict implementation success.
- Competence describes an increased confidence and self-efficacy, driven by technical knowledge acquisition. In this case, merging traditional and biomedical roles enhanced legitimacy as HIV supporters and strengthened connection to the formal health system.
- Self-determination reflects perceived autonomy. In contrast to assumptions that intervention fidelity must come at the expense of autonomy, we suggest that empowering implementers to adapt their roles within the bounds of core intervention components may reinforce engagement and create a sense of ownership.
- Impact relates to the perception or experience that the intervention will lead to a tangible benefit for the target population. This may act as a motivational reinforcement loop that sustains implementation of TSS even in the face of resource or structural constraints.
- External validation, the novel domain identified in this study, underscores the critical role of recognition from actors in the formal healthcare sector as part of empowerment. Echoing legitimacy theory, which posits that perceived institutional recognition enhances identity coherence and promotes role stability [58], external validation legitimized the work of THs within the broader health system. This may be particularly important for lay providers who have been historically excluded from institutional structures, or are members of a stigmatized class [59–61].

**Fig. 1:**
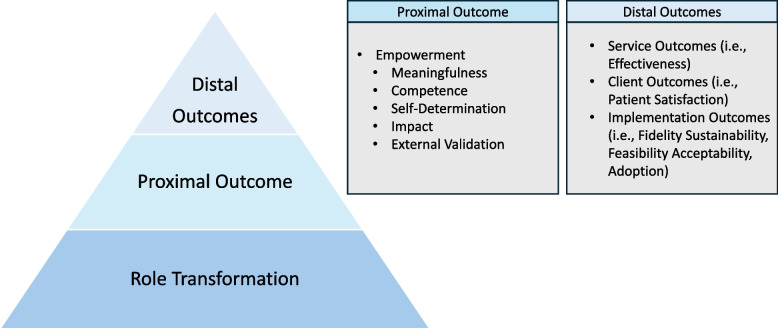
Summary of empowerment as a proximal implementation outcome, bridging the process of role transformation in TSS programs with broad distal outcomes of interest

Together, these five empowerment domains are integral to the process of role transformation as part of a TSS program. THs began to see themselves not only as traditional caregivers but as hybrid providers who could confidently navigate both cultural and biomedical realms. This identity shift is foundational to the domains of empowerment (Fig. 1). Our qualitative data suggest that as providers transform their roles, the domains of empowerment can facilitate intervention adoption, fidelity and maintenance. Conversely, the absence or erosion of these empowerment elements—such as limited role alignment, inadequate training, constrained autonomy, minimal perceived impact, or lack of recognition from biomedical actors—could function as barriers to implementation by weakening trust, motivation, and engagement. This suggests that empowerment is not only a positive result of participation, but could be considered a necessary condition for sustaining role transformation and effective task-shifting.

## Discussion

As global health systems confront persistent workforce shortages and structural barriers to equitable care, task shifting and task sharing (TSS) continue to be widely endorsed implementation strategies for closing health service gaps, particularly in resource-limited settings [6, 15]. However, evaluating the success of this strategy with informal lay providers such as traditional healers remains underexplored, both conceptually and operationally. This study addresses that gap by identifying empowerment as a proximal implementation outcome can indicate successful uptake, engagement, and sustainment of TSS interventions.

A critical gap identified in implementation science concerns the relationship between implementation strategies and implementation outcomes – particularly how strategies generate measurable changes that predict successful adoption, fidelity, and sustainability. Proctor’s 2011 and 2023 [47, 56] research agendas explicitly called for theory-building studies that use implementation outcomes to model mechanisms of implementation success [47], yet few studies examine these linkages [56]. Our study responds to this gap by proposing that empowerment—particularly among informal, community-based implementers—serves as both a proximal outcome and a passthrough mechanism that potentiates the success of TSS interventions.

By applying Lee and Koh’s empowerment framework [54] and expanding upon it with the novel concept of external validation, we conceptualize empowerment as a multi-dimensional construct comprising meaningfulness, competence, self-determination, impact, and recognition. This approach aligns with foundational public health frameworks, including the Alma Ata Declaration and Ottawa Charter, which identify community empowerment as central to equitable, sustainable health system development [62–64]. Our findings position these dimensions as proximal implementation outcomes as they not only reflect early indicators of implementation progress (i.e. adoption, fidelity), but also illuminate the mechanisms by which task shifting exerts its effects.

In line with Proctor’s emphasis on user-centered implementation outcomes [47], we frame empowerment as a psychologically grounded construct that connects the implementation strategy of TSS to behavioral change. This builds on emerging literature recognizing internal states—such as self-efficacy, perceived legitimacy, and motivation—as central to successful implementation [65–67]. While our study identified the proximal outcome of empowerment in light of a TSS program, we believe that role transformation is an integral component of any implementation strategy that involves training and knowledge transfer, including common strategies such as train-the-trainer and identify/prepare champions. Therefore, empowerment could be a broadly relevant proximal outcome for other types of implementation research.

Our findings also offer several implications for implementation programs involving the lay cadre:

### Target empowerment explicitly

Empowerment should be positioned as an explicit goal rather than a secondary outcome. While often conflated, motivation and empowerment are conceptually distinct. Motivation may yield short-term engagement, but empowerment—by integrating personal fulfilment with organizational purpose—supports distal implementation outcomes [52]. Training approaches should be designed intentionally to intentionally foster meaningfulness, competence, autonomy, perceived impact, and acceptability by institutional stakeholders— not only as side effects but as core targets of implementation efforts.

### Embed mechanisms of formal recognition

To ensure sustainability, consider strategies that involve formal recognition such as certificates, stipends, public acknowledgment, or structured inclusion in care coordination. These are not merely symbolic gestures; they directly address historical exclusion and help legitimize the roles of informal providers within the health system and contribute towards empowerment.

### Measure empowerment systematically

Capturing empowerment outcomes requires measurement approaches that reflect their experiential and identity-based nature. Unlike traditional outcomes—such as fidelity or feasibility—which are typically assessed through system-level indicators, empowerment outcomes are best evaluated through qualitative inquiry or tailored psychometric tools. Developing rapid-assessment instruments could enable implementers to detect early signs of disengagement and adjust strategies accordingly [68].

### Bridge frameworks to connect individual and system-level change

Our findings suggest that empowerment outcomes complement—but do not replace—established implementation outcome frameworks such as Proctor’s outcomes, CFIR and COM-B [33, 47, 56, 69]. Whereas Proctor's outcomes focus primarily on organizational performance and intervention-level metrics (e.g., feasibility, fidelity, adoption), empowerment outcomes highlight individual-level psychological and social mechanisms through which implementation strategies exert their effects. This multi-level integration advances calls for frameworks that bridge individual, organizational, and systemic levels of implementation [70, 71].

We note a few limitations of this qualitative study. Interviews were conducted immediately following the initial healer training and focused on participant motivation to participate in the study rather than the effectiveness of healers in their TSS role. Future work can understand how participant motivation and empowerment correspond with clinical effectiveness over the course of the study. Our findings capture early experiences of role transformation rather than long-term integration of new HIV-support responsibilities with traditional practices. Future research during the implementation and post-implementation phases of our study will explore how these roles evolve and become embedded in day-to-day healer practice. We also note that our characterization of empowerment reflects a self-assessed outcome measure. The broader utility of this new implementation outcome could be assessed through future research on quantifying the domains of empowerment, similar to how the Theoretical Framework of Acceptability evaluates components of acceptability [72].

## Conclusion

Our findings advance implementation science by proposing a refined, multi-dimensional conceptualization of empowerment as a proximal implementation outcome leading to successful TSS. We present a theory-driven analysis of qualitative data of traditional healers’ experiences following HIV lay supporter training illustrate five interrelated empowerment domains—meaningfulness, competence, self-determination, impact, and external validation. By adopting a user-centered perspective, this study contributes to expanding our understanding of implementation outcomes and deepening our understanding of how the strategy of task-shifting achieves its intended effects. Empowerment as a proximal implementation outcome suggests an important indicator for assessing and optimizing task shifting or other strategies involving informal cadres. Future research is needed to potentially validate quantitative measures of empowerment outcomes across diverse contexts and examine their predictive value in relation to traditional implementation outcomes such as adoption, fidelity, and sustainability.

## Data Availability

The datasets generated and analyzed during this study are not publicly available due to confidentiality agreements with participants but are available from the corresponding author upon reasonable request.

## Abbreviations

ART: Antiretroviral Therapy
CHW: Community Health Worker
HIV: Human Immunodeficiency Virus
IRB: Institutional Review Board
PLWH: People Living With HIV
TH: Traditional Healer(s)
TSS: Task Shifting and Task Sharing
UNAIDS: Joint United Nations Programme on HIV/AIDS
